# Cocaine Use and Incarceration: A Rare Cause of Bowel Ischemia, Perforation, and Gastrointestinal Hemorrhage

**DOI:** 10.7759/cureus.28538

**Published:** 2022-08-29

**Authors:** Pavan K Naidu, Lexi R Frankel, Summer Roorda, Michael Renda, Mark G Mckenney

**Affiliations:** 1 General Surgery, Kendall Regional Medical Center, Miami, USA; 2 College of Allopathic Medicine, Nova Southeastern University Dr. Kiran C. Patel College of Allopathic Medicine, Davie, USA; 3 General Surgery, Nova Southeastern University Dr. Kiran C. Patel College of Allopathic Medicine, Davie, USA

**Keywords:** trauma, crack-cocaine, gastrointestinal hemorrhage, perforation, bowel ischemia

## Abstract

Cocaine use is rising in persons ≥50 years old and in black and socioeconomically disadvantaged communities. Cocaine-induced bowel ischemia and gastrointestinal injury are deadly findings that have been previously described in the literature. In this report, we present a case of small bowel ischemia, perforation, and upper gastrointestinal hemorrhage co-occurring in a 62-year-old incarcerated male with a 15-year history of cocaine use. The patient presented from jail, peritonitic in septic shock, and was promptly taken for emergent surgical exploration. He was found to have massive fecal peritonitis secondary to full-thickness ischemia and perforation of the jejunum and ileum. Immediately postoperatively, the patient developed a large volume of hemorrhage from multiple gastric and duodenal ulcers refractory to endoscopic intervention, ultimately requiring emergent embolization of the gastroduodenal artery. His course was further complicated by severe septic shock with a blunted response to catecholamine vasopressors. Early recognition and aggressive treatment of the gastrointestinal complications and the unique critical care challenges associated with cocaine use facilitated this patient’s eventual full recovery.

## Introduction

Recent epidemiological data suggest an increase in global cocaine use [1]. Acute cocaine toxicity and long-term use can lead to life-threatening complications such as acute coronary syndrome, stroke, renal failure, and death [2]. Rarely, cocaine use can lead to gastrointestinal complications, including bowel ischemia, bowel perforation, and gastrointestinal hemorrhage [3].

While early recognition of cocaine-induced gastrointestinal injury is essential for a favorable prognosis, inmates often suffer from delayed medical care. Furthermore, ill-equipped correctional facilities, violence, and overcrowding lead to increased rates of medical complications, substance use, and addiction [4].

## Case presentation

**Figure 1:**
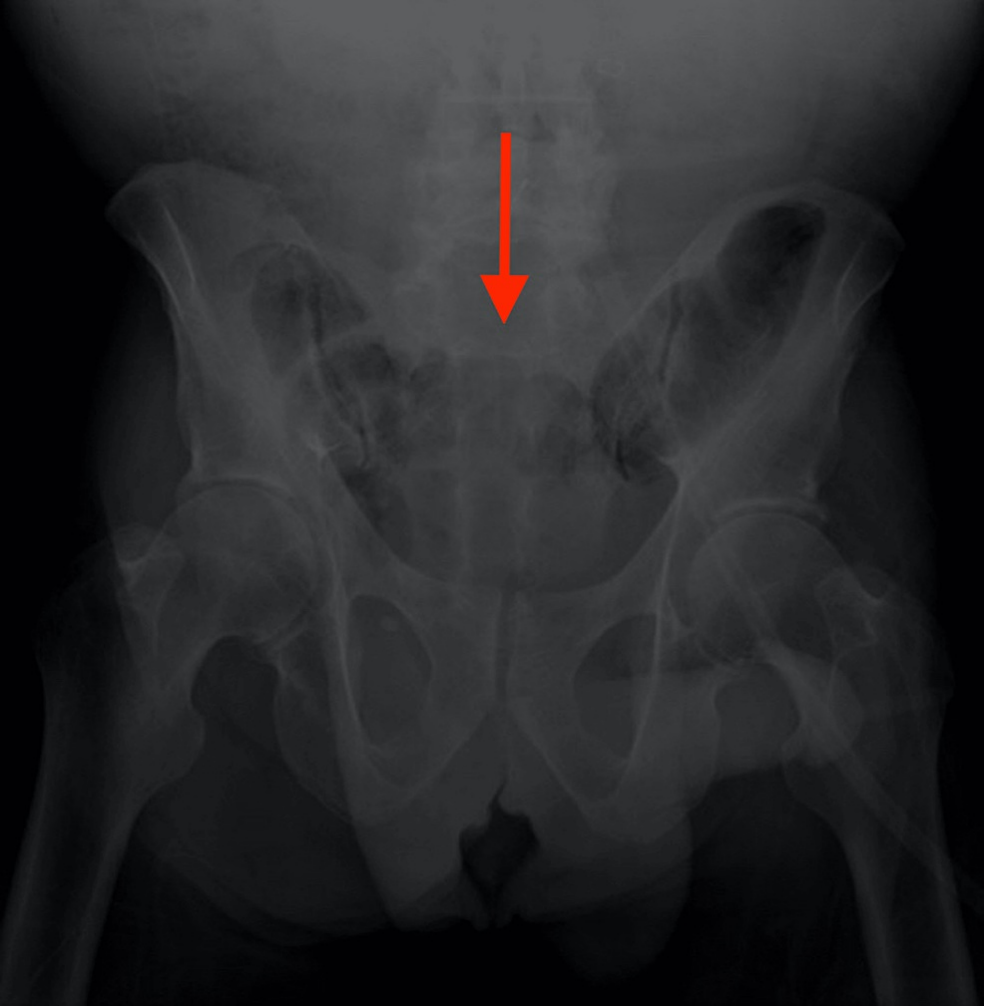
KUB demonstrating dilated small bowel loops suggestive of ileus or obstruction. This imaging does not provide definitive evidence of perforation. KUB: kidney, ureter, and bladder study.

A 62-year-old homeless incarcerated black male presented to the emergency room with four hours of sudden onset severe abdominal pain and coffee-ground emesis following two weeks of moderate diffuse abdominal pain. Previous medical histories include hypertension, gastroesophageal reflux disease (GERD), alcohol dependence, cirrhosis, and chronic crack cocaine use. The patient endorsed smoking crack cocaine the day prior to the presentation. The patient arrived tachycardic with mean arterial pressures below 40, unresponsive to dopamine drip and fluid resuscitation initiated by emergency medical services (EMS). An upper gastrointestinal bleed (UGIB) was suspected upon initial evaluation in the emergency department. Treatment was initiated with intravenous pantoprazole and resuscitation was continued with packed red blood cells and crystalloid fluids. A bedside point-of-care ultrasound revealed free fluid in the right upper quadrant and a distended small bowel. An abdominal radiograph revealed dilated small bowel loops but no definitive evidence of perforation (Figure 1). Worsening hypotension refractory to vasopressors and fluid resuscitation prompted the emergency physician to consult the acute care surgery (ACS) team. A massive transfusion protocol was initiated by ACS, and the patient was taken to the operating room for an emergent exploratory laparotomy. Upon reaching the abdominal cavity, dense adhesions and feculent peritonitis were encountered. Further exploration revealed a diffusely dilated small intestine with patchy areas of full-thickness ischemia and multiple perforations of the ileum and jejunum. Extensive enterolysis and resection of 50 cm of the small intestine were performed. The patient’s worsening hemodynamic status, hypothermia, and onset of coagulopathy prompted the surgical team to leave the bowel in discontinuity with a negative pressure abdominal wound vacuum for temporary closure. The patient was transferred to the surgical intensive care unit (SICU) for further resuscitation.

**Figure 2:**
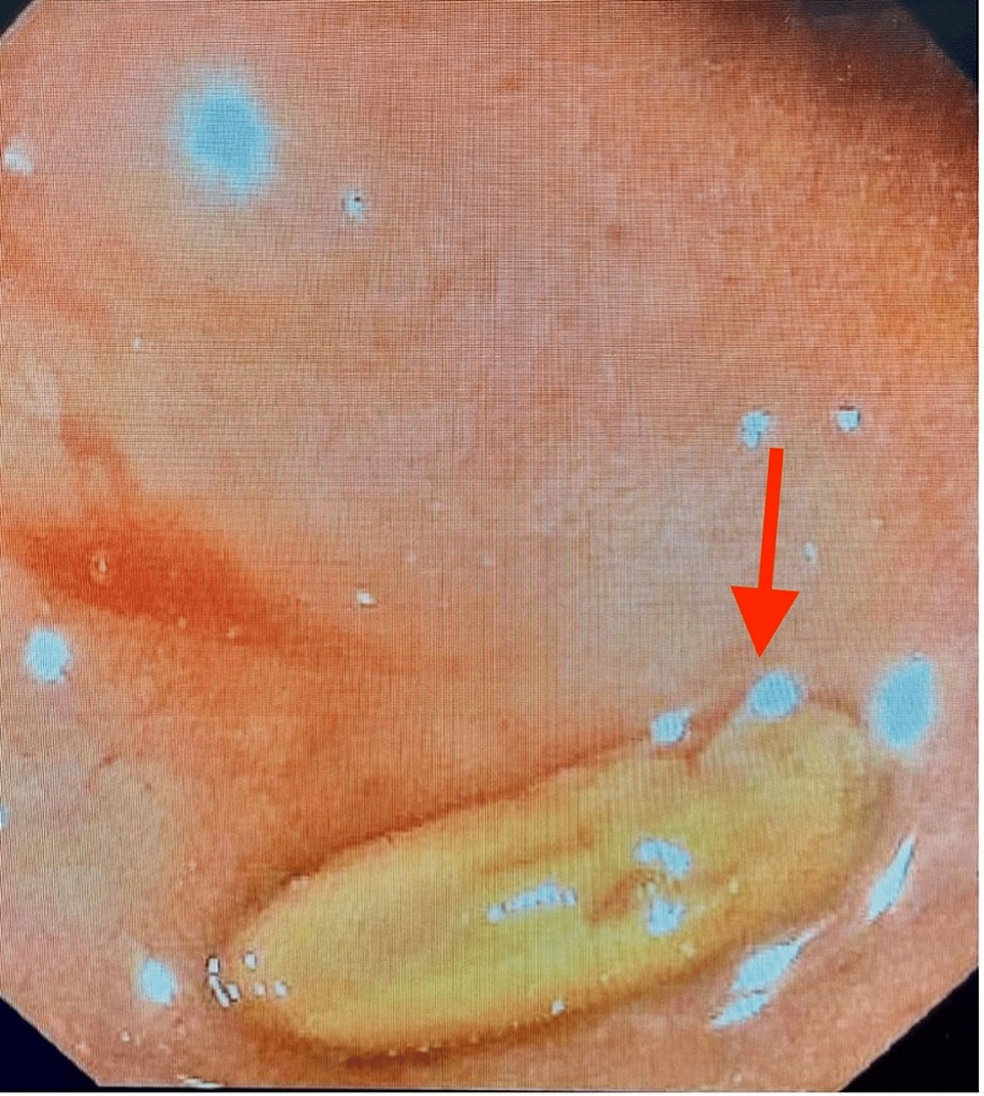
EGD image revealing nonbleeding cratered ulcer in the duodenal bulb. EGD: esophagogastroduodenoscopy.

**Figure 3:**
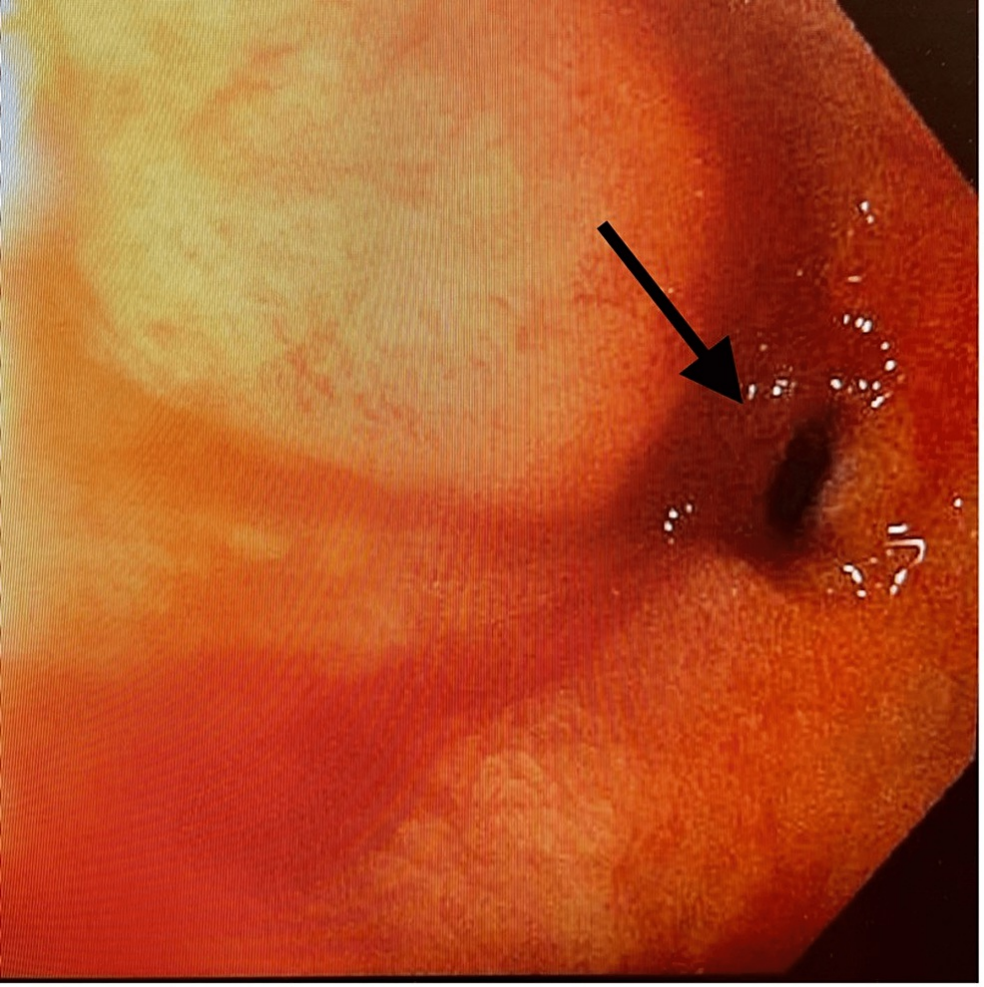
EGD image exposing severe stenosis of the first portion of the duodenum. EGD: esophagogastroduodenoscopy.

**Figure 4:**
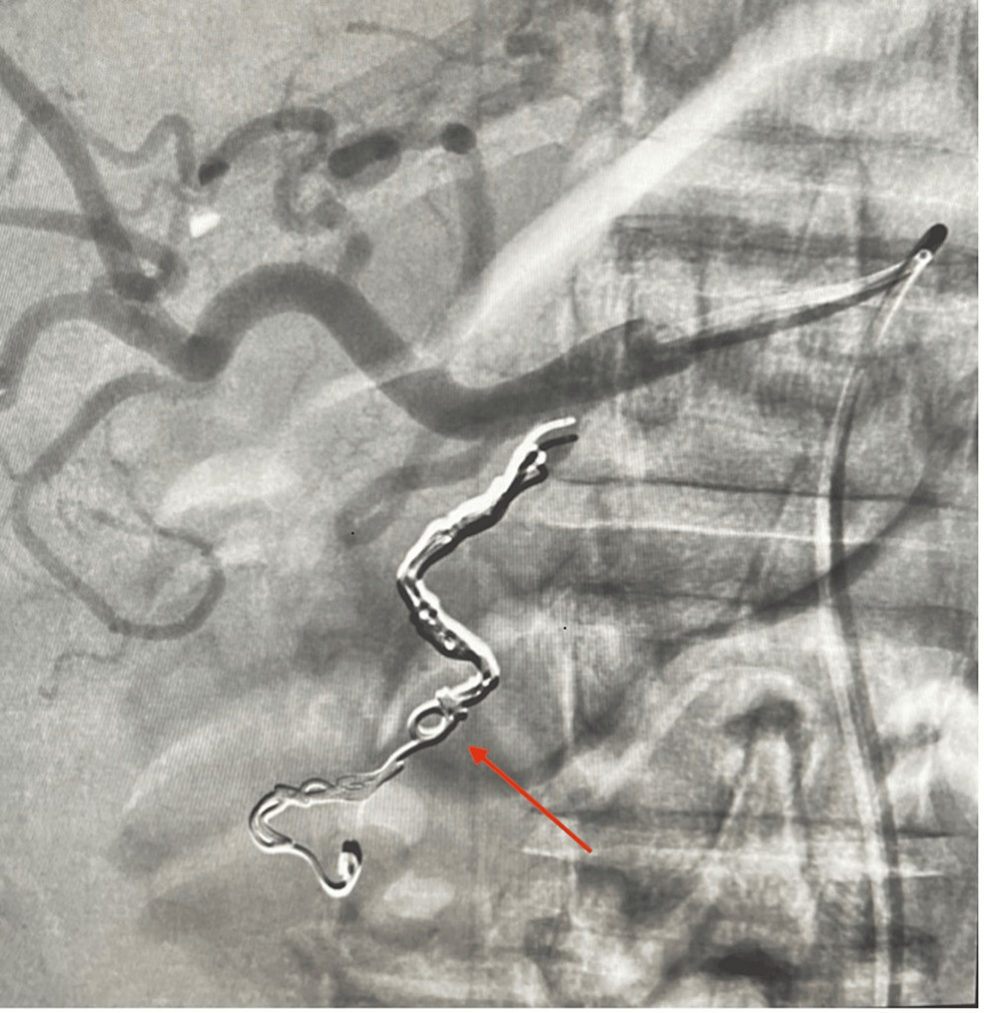
Digital subtraction angiography demonstrating embolization of the gastroduodenal artery using a series of 0.035 inch metallic coils.

**Figure 5:**
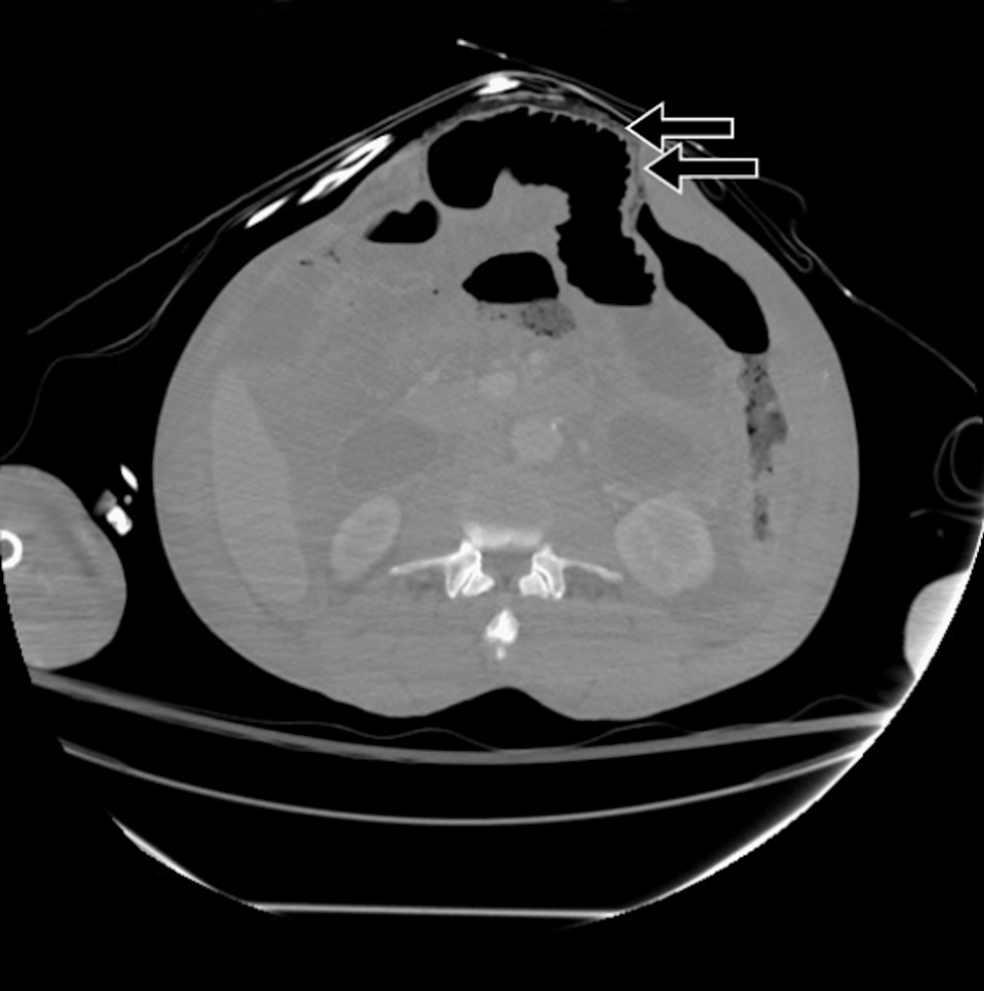
Abdomen/pelvis CT demonstrating pneumatosis of the small bowel, suggestive of ischemia (arrows).

**Figure 6:**
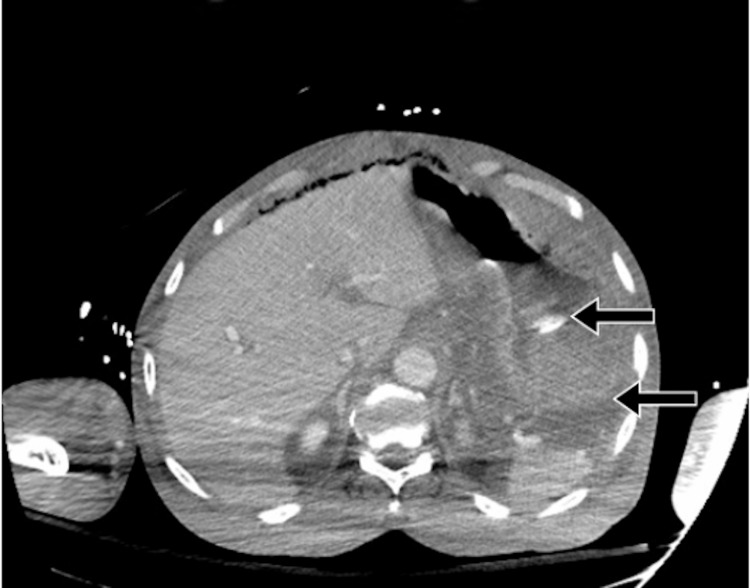
Abdomen/pelvis CT revealing hyperdense fluid in the stomach and OG tube in place, consistent with upper GI bleed (arrows).

On arrival at the SICU, 1500 mL of bright red blood was evacuated from the orogastric tube. Pantoprazole and octreotide drips were started, and a massive transfusion protocol was continued. The gastroenterology team was called for a bedside emergent endoscopic intervention. Elevated peak airway pressures gave suspicion for abdominal compartment syndrome, which was confirmed by measurement of bladder pressures between 22 and 25 mmHg. The negative pressure wound vacuum was released, causing an improvement in his hemodynamic status and bladder pressure. Esophagogastroduodenoscopy (EGD) was then performed. A prepyloric gastric ulcer was visualized and a hemoclip was applied. A second non-bleeding ulcer was visualized in the duodenal bulb immediately distal to the pylorus (Figure 2). The first portion of the duodenum was severely stenosed at the site of a chronic ulcer with ongoing bleeding visualized distal to the obstruction. The endoscope was unable to traverse this stenosis (Figure 3). Interventional radiology was then consulted for emergent angioembolization. An angiogram revealed engorgement of the right gastric artery and arterial blush in the first and second portions of the duodenum. Embolization of the gastroduodenal artery was performed, significantly improving overall hemodynamic status (Figure 4). Subsequent CT demonstrated pneumatosis in the small bowel consistent with ischemia and hyperdense fluid in the stomach (Figure 5), suggestive of residual gastric bleeding (Figure 6).

Severe septic shock with blunted responses to multiple catecholamine vasopressors delayed the patient’s return to the operating room in the following days. Despite the resolution of initial lactic acidosis with fluid resuscitation and a trial of stress-dose hydrocortisone, the patient continued to require very high doses of multiple vasopressor drips. He was found to be highly responsive to dopamine allowing us to wean norepinephrine, vasopressin, and epinephrine drips by day 3 postoperatively. Then, on day 4, the patient was taken back for a repeat exploratory laparotomy. The well-perfused, viable-appearing small bowel was noted without signs of leakage or necrosis at the staple lines. A stapled side-to-side antiperistaltic anastomosis was created between the jejunum and distal ileum. Despite minimal blood loss, the patient became increasingly hypotensive, requiring high doses of multiple vasopressors. The decision was made to delay abdominal closure, and a negative-pressure abdominal wound vacuum was used for temporary closure. The patient underwent a third exploratory laparotomy one week after his initial presentation, revealing areas of patchy ischemic necrosis of the proximal jejunum without frank perforation. Eight centimeters of proximal jejunum was resected and a stapled side-to-side antiperistaltic anastomosis was created. The patient was hemodynamically stable at this time, and the abdominal skin and fascia were closed. The patient made a full recovery over seven weeks and was ambulatory with normal bowel function on discharge.

## Discussion

Cocaine-induced bowel ischemia, perforation, and gastrointestinal hemorrhage have been described in scarce reports in the literature [3,5-8]. Gastrointestinal complications resulting from cocaine abuse are rare, and the simultaneous occurrence of three such complications occurs even more infrequently. To the best of our knowledge, this is the first report to describe cocaine-induced bowel ischemia, perforation, and upper gastrointestinal hemorrhage presenting concurrently.

The pathophysiology of cocaine-induced ischemia is explained via the inhibition of norepinephrine reuptake at presynaptic terminals, leading to its collection at postsynaptic terminals [3]. This accumulation of catecholamines causes extensive vasoconstriction and ischemia and can lead to potentially lethal complications such as gastrointestinal perforation and ulceration, acute coronary syndromes, stroke, and thrombosis [3].

Various historical and environmental factors may have contributed to this patient’s unusual presentation. The patient’s ten-year history of GERD likely heightened his susceptibility to developing gastrointestinal ulcers [9], while his incarceration likely limited his access to antacids that he reported taking as needed prior to entering the correctional facility. Additionally, substance use is significantly increased in incarcerated people while healthcare quality is significantly decreased [10]. The culture of correctional facilities often creates an environment of limited communication between healthcare providers and custody staff, leading to medical errors and delays in treatment [10]. The patient presented in this report had been complaining of worsening abdominal pain for two weeks and sudden onset of severe abdominal pain and vomiting for four hours before being transported to the emergency department.

## Conclusions

Improved communication between healthcare providers and correctional officers is crucial for early detection and prompt treatment of the acute abdomen in incarcerated persons. As the incidence of cocaine use continues to rise, acute complications such as bowel perforation and gastrointestinal hemorrhage pose significant time-contingent diagnostic dilemmas. Cocaine-induced gastrointestinal injury, especially in the setting of incarceration, is a life-threatening disease that requires rapid diagnosis and treatment to preclude invasive surgical management, extensive length of stay, and preventable long-term complications.

## References

[1] John WS, Wu LT (2017). Trends and correlates of cocaine use and cocaine use disorder in the United States from 2011 to 2015. Drug Alcohol Depend.

[2] Riezzo I, Fiore C, De Carlo D, Pascale N, Neri M, Turillazzi E, Fineschi V (2012). Side effects of cocaine abuse: multiorgan toxicity and pathological consequences. Curr Med Chem.

[3] Tiwari A, Moghal M, Meleagros L (2006). Life threatening abdominal complications following cocaine abuse. J R Soc Med.

[4] Massoglia M, Pridemore WA (2015). Incarceration and health. Annu Rev Sociol.

[5] Linder JD, Mönkemüller KE, Raijman I, Johnson L, Lazenby AJ, Wilcox CM (2000). Cocaine-associated ischemic colitis. South Med J.

[6] Scharff JR, Longo WE, Vartanian SM, Jacobs DL, Bahadursingh AN, Kaminski DL (2003). Ischemic colitis: spectrum of disease and outcome. Surgery.

[7] Bansal R, Sharma M, Aron J (2019). Cocaine gut. ACG Case Rep J.

[8] Carlin N, Nguyen N, DePasquale JR (2014). Multiple gastrointestinal complications of crack cocaine abuse. Case Rep Med.

[9] Tang RS, Wu JC (2013). Managing peptic ulcer and gastroesophageal reflux disease in elderly Chinese patients--focus on esomeprazole. Clin Interv Aging.

[10] Wilper AP, Woolhandler S, Boyd JW, Lasser KE, McCormick D, Bor DH, Himmelstein DU (2009). The health and health care of US prisoners: results of a nationwide survey. Am J Public Health.

